# Citral protects against metabolic endotoxemia, and systemic disorders caused by high-fat diet-induced obesity via intestinal modulation

**DOI:** 10.3389/fphar.2025.1567217

**Published:** 2025-04-07

**Authors:** Maycon Tavares Emílio-Silva, Vinicius Peixoto Rodrigues, Mariana Moraes Fioravanti, Antonio Jesús Ruiz-Malagon, Matheus Naia Fioretto, Priscila Romano Raimundo, Rie Ohara, Renata Assunção, Gabriela Bueno, Felipe Lima Dario, Luis Antonio Justulin, Alba Rodríguez-Nogales, Lucia Regina Machado da Rocha, Júlio Gálvez, Clélia Akiko Hiruma-Lima

**Affiliations:** ^1^ Department of Structural and Functional Biology, Physiology Sector, Institute of Bioscience, São Paulo State University (UNESP), Botucatu, Brazil; ^2^ Department of Pharmacology, Center for Biomedical Research (CIBM), University of Granada, Granada, Spain; ^3^ Department of Structural and Functional Biology, Morphology Sector, Institute of Bioscience, São Paulo State University, (UNESP), Botucatu, Brazil; ^4^ CIBER de Enfermedades Hepáticas y Digestivas (CIBER-EHD), Instituto de Salud Carlos III, Madrid, Spain

**Keywords:** systemic inflammation, monoterpene, lipopolysaccharide, tight junctions, dyslipidemia

## Abstract

**Background:**

Obesity is a growing global epidemic associated with changes in the gut microenvironment and metabolic endotoxemia, which can exacerbate metabolic and inflammatory processes. Citral (CT), a monoterpene present in essential oils, has been investigated for its anti-inflammatory, antioxidant, and immunomodulatory properties. However, its role in modulating the gut axis during metabolic and inflammatory alterations in obesity remains unknown. In this study, we investigated the effects of CT on intestinal and metabolic impairment induced by lipopolysaccharide (LPS) and high-fat diet (HFD) in *in vitro* and *in vivo* models.

**Methods:**

Male C57BL/6J mice were fed a standard diet and HFD for 17 weeks, with daily oral administration of CT treatment (25, 100, or 300 mg/kg) or vehicle. Morphological and histological parameters, lipid profiles, adipose index, cytokine levels, and colonic gene expression were determined. *In vitro*, murine rectal carcinoma (CMT-93) cells were stimulated with LPS (10 μg/mL) to assess tight junction and inflammatory protein expression.

**Results:**

CT treatment showed anti-obesity activity against HFD-induced body mass gain in mice, which was attributed to a significant reduction in body fat, glycemia, and cholesterol levels. Systemic inflammation during obesity also decreased after CT treatment, with a significant reduction in serum levels of endotoxin, interleukin-1β, and tumor necrosis factor-α. Additionally, CT stimulation reduced inducible nitric oxide synthase expression and maintained ZO-1 levels in LPS-stimulated CMT-93 cells.

**Conclusion:**

CT has anti-obesogenic, anti-inflammatory, and anti-hyperlipidemic properties mediated by its protective effects on the intestinal epithelium in obesity. Thus, our results highlight the promising preclinical results of CT treatment as a protective agent against the detrimental effects of HFD and LPS in mice.

## 1 Introduction

In 2022, the World Health Organization (WHO) estimated one billion people worldwide to be obese, of which 650 million were adults and 370 million were adolescents and children (WHO - World Health Organization, 2022). In the absence of intervention, an alarming global scenario is predicted for 2050, with an estimated 3.80 billion individuals expected to be overweight and obese (Ng et al., 2025). Obesity is defined as an increase in body weight due to the accumulation of lipids in the subcutaneous and visceral adipose tissue (AT), with ectopic deposition of lipids in other tissues (Calder et al., 2011; Turpin et al., 2009). Additionally, obesity is directly associated with an imbalance in the gut environment. In this context, dysbiosis of the intestinal microbiota leads to dysfunction of the integrity of the gut barrier in obese individuals (Rosendo-Silva et al., 2023). Consequently, there is an increase in the permeability of the mucosa, with the activation of the immune system triggering an inflammatory process, leading to the development of intestinal and systemic diseases (Turner, 2009; Acciarino et al., 2024).

The ingestion of a high-fat diet (HFD) induces an inflammatory response with the activation of nuclear factor kappa B, resulting in the expression of NLRP-3, interleukin 6 (IL-6), and inducible nitric oxide synthase (iNOS) in the gastrointestinal tract (Ding et al., 2010). This response, associated with an unbalanced diet, impairs the expression of NLRP-6 and tight junction proteins (TJ) in the gut, thereby facilitating the increased passage of lipopolysaccharide (LPS) from the lumen into the circulation in obese individuals (Cani et al., 2012; Kumar et al., 2024). Consequently, obese individuals have an increased concentration of systemic LPS, known as metabolic endotoxemia (Cani et al., 2007; Ghosh et al., 2020).

Metabolic endotoxemia promotes changes in the levels of systemic inflammatory mediators and causes the development of metabolic disorders, including, but not limited to, hyperglycemia, hypertriglyceridemia, dyslipidemia and hypertension (Boulangé et al., 2016). Given the increasing incidence and prevalence of obesity in society, the search for pharmacological alternatives is necessary to reduce the costs of existing therapeutic options and minimize side effects (Saunders et al., 2018; Kushner, 2014). However, owing to the multifactorial nature of obesity, its treatment requires combined approaches, such as lifestyle changes, dietary re-education, and physical exercise. Moreover, the use of pharmacological approaches is necessary; however, these strategies have limited proven therapeutic options and are ineffective in the long term, with significant adverse effects (Saunders et al., 2018; Prado et al., 2024).

Citral (CT) is a monoterpene mixture (neral and geranial) present in essential oils of different plant species, such as *Zingiber officinale* (ginger) and *Cymbopogon citratus* (lemongrass) (Mangprayool et al., 2013; Nishijima et al., 2014). CT has anti-inflammatory, antipyretic, antibacterial, antitumor, and analgesic properties which have been demonstrated in several experimental models (Sharma et al., 2019). Additionally, previous studies have demonstrated the anti-obesity effect of CT in HFD-induced obese mice, which suppressed abdominal lipid accumulation, increased energy production by metabolism, and reduced serum levels of free fatty acids, triglycerides, and total cholesterol (Modak and Mukhopadhaya, 2011). Furthermore, Lai and colleagues demonstrated the hepatoprotective effect of monoterpene, which exhibited protective activity against HFD-induced liver damage in mice, mediated by the reduction of oxidative stress and inflammatory processes (Lai et al., 2016). CT treatment also exhibits an anti-inflammatory effect in obese mice when challenged with LPS by reducing serum levels of tumor necrosis factor (TNF)-α and leptin (Emílio-Silva et al., 2020). Previously, our group also observed that CT promotes the healing of acetic acid-induced gastric lesions in obese mice (Ohara et al., 2023). Despite the well-documented pharmacological actions of CT, its effects on metabolic endotoxemia and intestinal changes associated with obesity remain unknown. Thus, we aimed to investigate the effect of chronic CT treatment on the metabolic and inflammatory changes associated with LPS and HFD in *vitro* and *in vivo* models that mimic obesity.

## 2 Materials and methods

### 2.1 *In vivo* experiments

#### 2.1.1 Animal

All experiments were approved by the Ethics Committee for Animal Use of the Institute of Biosciences (process number 6702310820). All experiments were performed in accordance with the Brazilian legislation regulated by the National Council for the Control of Animal Experimentation (CONCEA) and the Ethical Principles in Animal Research formulated by the Brazilian Society of Science in Laboratory Animals. Male C57Bl/J6 mice (5-6 weeks old) were purchased from the Multidisciplinary Center for Research on Laboratory Animals/Unicamp (Campinas, Brazil). The animals were maintained in cages of 10 animals, under controlled temperature (28 ± 1°C) and a 12/12-hour light-dark cycle, with filtered water and food *ad libitum*.

**FIGURE 1:**
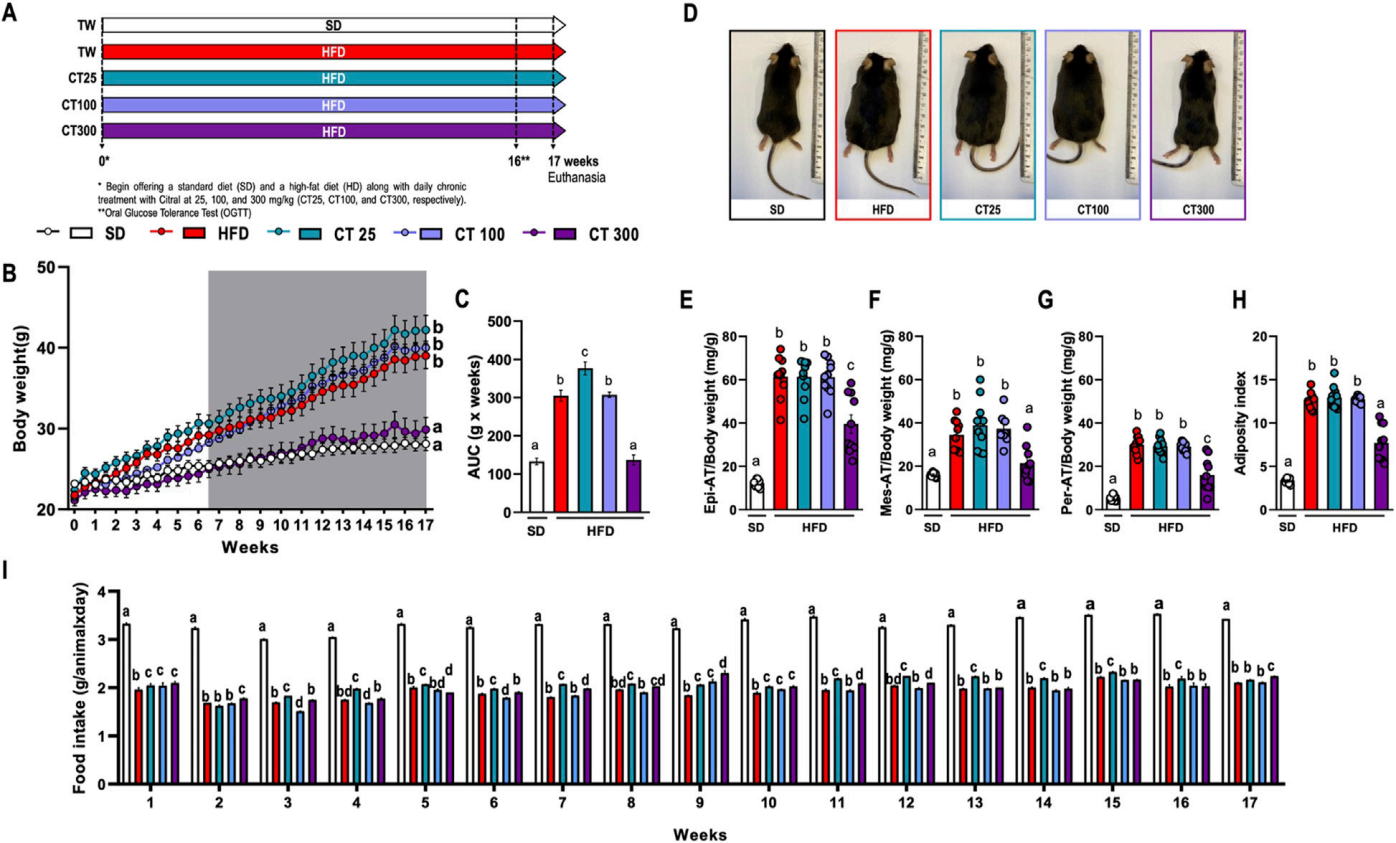
Impact of chronic Citral (CT; 25, 100 and 300 mg/kg) treatment following 17 weeks of a high-fat diet-induced obesity in C57Bl/J6 mice. **(A)** Experimental design. **(B)** Evolution of body weight. **(C)** Area under the curve (AUC) of body weight development over 17 weeks. **(D)** Representative images of each experimental group. Relative mass of epididymal [Epi; **(E)**], mesenteric [Mes; **(F)**], and peritoneal [Per; **(G)**] AT. **(H)** Adiposity index of the sum of all AT. **(I)** Food intake by group over 17 weeks. Data are expressed as mean ± S.E.M (n = 10). Different lowercase letters (e.g., “a,” “b,” “c,” and “d”) indicate statistically significant differences among the groups (*p* < 0.05), with different letters representing distinct groups and shared letters indicating no significant difference. SD, standard diet; HFD, high-fat diet.

After 1 week of adaptation, the mice were divided into five groups (n = 10 per group) according to the treatment and diet received: standard diet (SD; Presence, Paulínia, SP, Brazil) with vehicle (1% at Tween 80, 10 mL/kg, p.o.; Vetec, Rio de Janeiro, RJ, Brazil); and four treatment groups were fed high-fat diet (HFD; PragSoluções Biociências, Jaú, SP, Brazil) (Arçari et al., 2013). Each HFD group received daily oral (p.o.) administration of vehicle (obese control group) or CT (Sigma, St. Louis, MO, United States) at 25, 100, and 300 mg/kg (CT25, CT100 and CT300 groups, respectively) for 17 weeks (Figure 1A). Body weight and food intake were evaluated throughout the induction of obesity. In the 16th week, the animals were fasted, and the oral glucose tolerance test (OGTT) was performed. After 17 weeks, blood was collected and euthanasia via cardiac puncture was performed under anesthesia with isoflurane (induction: 5% and maintenance: 2%; Isoforine, Cristália, Itapira, São Paulo, Brazil). Samples were collected in a serum-separator tube for centrifugation (3000 × g, 15 min, 25°C) and serum was stored at −80°C. Epididymal (Epi), mesenteric (Mes), and peritoneal (Per) AT, colon tissues and fecal pellets from the colon were weighed and then stored at −80°C for biochemical and molecular analyses.

#### 2.1.2 OGTT

To investigate the effects of CT on peripheral glucose metabolism, the animals (n = 7 per group) were fasted for 6 h before testing. Blood samples were collected from the tail, and fasting blood glucose was determined using a glucose monitor (Accu-Chek Active Roche, Mannheim, Germany). Subsequently, the mice were administered an oral glucose solution (1 g/kg, p.o.), and blood glucose measurements were repeated at 15, 30, 60, and 120-min. The results were expressed as mg/dL.

#### 2.1.3 Histological procedures

Morphological and morphometric analyses were performed on colon samples fixed in methacarn (60% methanol/30% chloroform/10% acetic acid) for 4 h (Puchtler et al., 1985). The samples were dehydrated in a graded series of ethanol, diaphanized in xylene, and embedded in Paraplast (Sigma-Aldrich, St. Louis, MO, United States). Sections were cut at a 5 μm thickness on a microtome, collected on labeled slides and stored until they were used for morphometric analysis. Sections were stained with hematoxylin and eosin (H&E) and resorcin-fuchsin (Fuchsin) for elastin deposition, and three animals/slides were collected per experimental group (10 photomicrographs per slide/30 photomicrographs in total). All images were captured using a conventional LeicaDM2500 light microscope (Leica Microsystems, Nussloch, Germany) coupled with a digital camera and image analysis software (Leica Qwin.V3, Nussloch, Germany) at 20x and 40x magnification, respectively. Each microscopic field was analyzed using ImageJ 1.45 software (NIH, Bethesda, Maryland, United States).

#### 2.1.4 Protein quantification

Samples of AT and feces were homogenized in ice-cold phosphate buffer (0.01 M, pH 7.4) at a ratio of 1:4 (w/v). The homogenates were prepared using Polytron and centrifuged at 4°C for 5 min at 5,000 × g. The supernatant obtained was used to determine the total protein levels using Coomassie Plus™ Protein Assay Reagent (Thermo Fisher Scientific, Unites States) following the manufacturer’s instructions. Results were expressed as μg/mL.

#### 2.1.5 Fecal evaluation

Fecal pellets were suspended in a buffer solution containing 10 mM Tris-HCl, 1 mM MgCl_2_, and 1 mM ZnCl_2_, pH 8.0, with the addition of 1% protease inhibitor cocktail (PIC; Sigma, St. Louis, MO, Unites States) and processed as described by Toivio et al. (2023). Intestinal alkaline phosphatase activity and total cholesterol levels in fecal samples were measured using biochemical kits (REF 79-4/30 and REF 76-2/100, respectively; Labtest Diagnóstico S.A., MG, Brazil). Results were expressed as µM and mg/μg of protein, respectively.

#### 2.1.6 Quantitative RT-PCR

Total RNA was extracted from the colon samples using Brazol reagent (LGC Biotenologia, Cotia, SP, Brazil). A High-Capacity complementary DNA (cDNA) Reverse Transcription Kit (Applied Biosystems, Foster City, Unites States) was used for cDNA synthesis. Quantitative PCR was performed using GoTaq qPCR Master Mix (Promega, Madison, WI, Unites States) and primers with the sequences listed in Table 1. The PCR was performed using Step One (Applied Biosystems, Foster City, CA, Unites States). The relative expression of target genes was calculated based on the threshold cycle (Ct) and normalized to β-actin and glyceraldehyde phosphate dehydrogenase (GAPDH) (Livak and Schmittgen, 2001).

**TABLE 1:** Primers sequence.

Gene	Sequence	T °C
*β-actin*	F: 5′- ATG​TGG​ATC​AGC​AAG​CAG​GAG -3′	60
	R: 5′- GGT​GTA​AAA​CGC​AGC​TCA​GTA​AC -3′	
*Gadph*	F: 5′- CTC​TCT​GCT​CCT​CCC​TGT​TC -3′	60
	R: 5′- CAA​ATC​CGT​TCA​CAC​CGA​CC -3′	
*Muc2*	F: 5′- GCA​GTC​CTC​AGT​GGC​ACC​TC-3′	61
	R: 5′- CAC​CGT​GGG​GCT​ACT​GGA​GAG-3′	
*Nrlp6*	F: 5′- GAG​GTT​CAG​GGA​CAG​GTC​CTA -3′	58
	R: 5′- CTC​AGC​ACT​CTC​AAG​CCA​CT -3′	
*Nrlp3*	F: 5′- TAT​GTT​GGA​CTG​GGC​ACT​GG -3′	53
	R: 5′- TAG​ACT​CCT​TGG​CGT​CCT​GA -3′	
*Tlr-4*	F: 5′- ACT​GGA​CAC​ATC​TTG​CCT​GG -3′	60
	R: 5′- AGG​TGT​CAT​GAA​GGC​TGG​TG -3′	
*Il-10*	F: 5′- AAA​AGG​TGC​CAC​CCT​GAA​GA -3′	60
	R: 5′- GAT​GTG​GTG​GGA​CCA​ACC​TT -3′	

#### 2.1.7 Endotoxin levels

Serum LPS levels associated with metabolic endotoxemia were determined using the Pierce™ Chromogenic Endotoxin Quant Kit (ThermoFisher Scientific, Unites States) according to the manufacturer’s instructions. The results were expressed as EU/mL.

#### 2.1.8 Serum inflammatory mediators’ levels

IL-1β and TNF-α serum levels were quantified using a Mouse Cytokine/Chemokine Magnetic Luminex Assay (KITL130871; R&D Systems, Inc., Minneapolis, MN, Unites States) with MAGPIX Luminex equipment (Milliplex MAP, Millipore, Burlington, Massachusetts, Unites States) following the manufacturer’s technical guidelines. Values were expressed as pg/mL.

#### 2.1.9 Lipid profile and TBARs quantification

Lipid profile markers were analyzed to determine the effects of the monoterpene on systemic lipid metabolism. The serum levels of total cholesterol (REF 76-2/100; Labtest Diagnóstico S.A., MG, Brazil), high-density lipoprotein (HDL; REF 13-50; Labtest Diagnóstico S.A., MG, Brazil) and triglycerides (REF 87-2/100; Labtest Diagnóstico S.A., MG, Brazil) were evaluated along with the quantification of very low-density lipoprotein (VLDL) and low-density lipoprotein (LDL), following the manufacturer’s technical guidelines.

$$VLDL\;cholesterol=\frac{Triglycerides}{5}$$

$$LDL\;Cholesterol=Total\;Cholesterol-\left(HDL+VLDL\right)$$

Lipid peroxidation in the serum and Epi AT samples was determined by the amount of malondialdehyde (MDA), a 2-thiobarbituric acid reactive species (TBARS). TBARS were extracted by dissolving thiobarbituric acid (TBA) in dimethyl sulfoxide (DMSO) and 10% (w/v) trichloroacetic acid in H_2_O and dissolving 1,1,3,3-tetramethoxypropane in ethanol. The absorbance was read at 535 nm and the results were expressed as µM/µg protein.

### 2.2 *In vitro* experiments

#### 2.2.1 Cell line

The CMT-93 rectal carcinoma cell line was obtained from the Cell Culture Unit at the University of Granada (Granada, Spain). Cells were cultured at 37ºC and 5% CO_2_ in DMEM (Dulbecco’s modified Eagle’s medium) supplemented with 10% fetal bovine serum, 2 mM glutamine, 1% penicillin/streptomycin, and 1% amphotericin B.

#### 2.2.2 MTT assay

Cell viability was evaluated using an MTT assay (Araújo et al., 2017; Green et al., 1982). The CMT-93 cells were seeded in 96-well plates and incubated with different concentrations of the CT (1, 5, 10, 25, 50, and 100 μg/μL) with 1% DMSO. After 3 h, all cells were stimulated with LPS (10 μg/mL) for 72 h. Each experimental group was prepared in quadruplicate.

After the stimulation period, the supernatant was removed, and 3-(4,5-dimethylthiazol-2-yl)-5-(3-carboxymethoxyphenyl)-2-(4-sulfophenyl)-2H-tetrazolium (MTS) solution was added to each well and incubated for 1 h in a cell viability assay. The absorbance of the supernatant was measured at 490 nm.

#### 2.2.3 Western blot assay

Proteins were extracted from LPS-stimulated CMT-93 cells treated with CT (5, 10, and 25 μg/mL) after 72 h. Each experiment was performed in triplicate. Protein concentrations were measured using the bicinchoninic acid colorimetric assay (BCA). Proteins were separated SDS-PAGE and were subsequently transferred onto a polyvinylidene fluoride membrane (GE Healthcare Life Sciences, Marlborough, MA, United States). After blocking with 5% milk, each membrane was incubated at 4°C overnight with primary antibodies: anti-iNOS (1:500 dilution), anti-ZO-1 (1:500 dilution) (Abcam, Cambridge, CB2 0AX, UK), anti-IL-6 (1:500 dilution), and anti-Occludin (1:1,000 dilution) (Life Technologies, Heidelberg, DE, United States). The membranes were then incubated with the respective secondary antibodies, anti-rabbit (1:5,000 dilution) and anti-mouse (1:3,000 dilution) (Sigma-Aldrich, Madrid, Spain), for 1 h at 25°C. β-actin (Santa Cruz Biotechnology, Inc., Heidelberg, Germany) at 1:1,000 dilution was used as an internal reference to all membranes. After incubation, the membranes were exposed to enhanced chemiluminescence kit for signal intensity quantification (Bio-Rad Laboratories, Madrid, ES, Spain). The obtained images were evaluated using ImageJ Fiji Software (Yang and Mahmood, 2012).

### 2.3 Statistical analysis

All statistical analyses were performed using GraphPad Prism (GraphPad Software, San Diego, CA, United States). All results are expressed as the mean ± standard error of the mean (SEM). Statistical analysis was performed using two-way analysis of variance (ANOVA) followed by Tukey’s test, and differences were considered significant at P < 0.05. In group comparisons, different lowercase letters (e.g., “a,” “b,”, “c,” and “d”) denote statistically significant differences, where groups sharing the same letter are not significantly different from each other, while those with different letters indicate significant differences.

## 3 Results

### 3.1 Citral prevents the obesity development of HFD-fed mice

Chronic ingestion of HFD caused a significant increase in body weight after 17 weeks of obesity induction (Figures 1B, C). This observation was further corroborated by analysis of representative images from the SD and HFD groups (Figure 1D). This increase was also observed in the relative weights of Epi- (Figure 1E), Mes- (Figure 1F), and Per-AT (Figure 1G) compared to those in the SD group (p < 0.05). The same weight increment profile was reflected in the adiposity index (Figure 1H), indicating the development of obesity in mice fed this diet.

However, daily treatment with CT at a dose of 300 mg/kg (CT300) prevented the body weight increase caused by the HFD, as evidenced by body weight evolution over the course of 17 weeks (Figures 1B, C). This response was associated with a significant reduction in the relative weight of AT and adiposity index compared to the control obese group (Figures 1E–H). However, despite the reduced food intake observed in HFD-fed animals, CT300 did not affect food intake over a 17-week period (Figure 1I).

### 3.2 Citral reduced the metabolic alterations in obese mice

**FIGURE 2:**
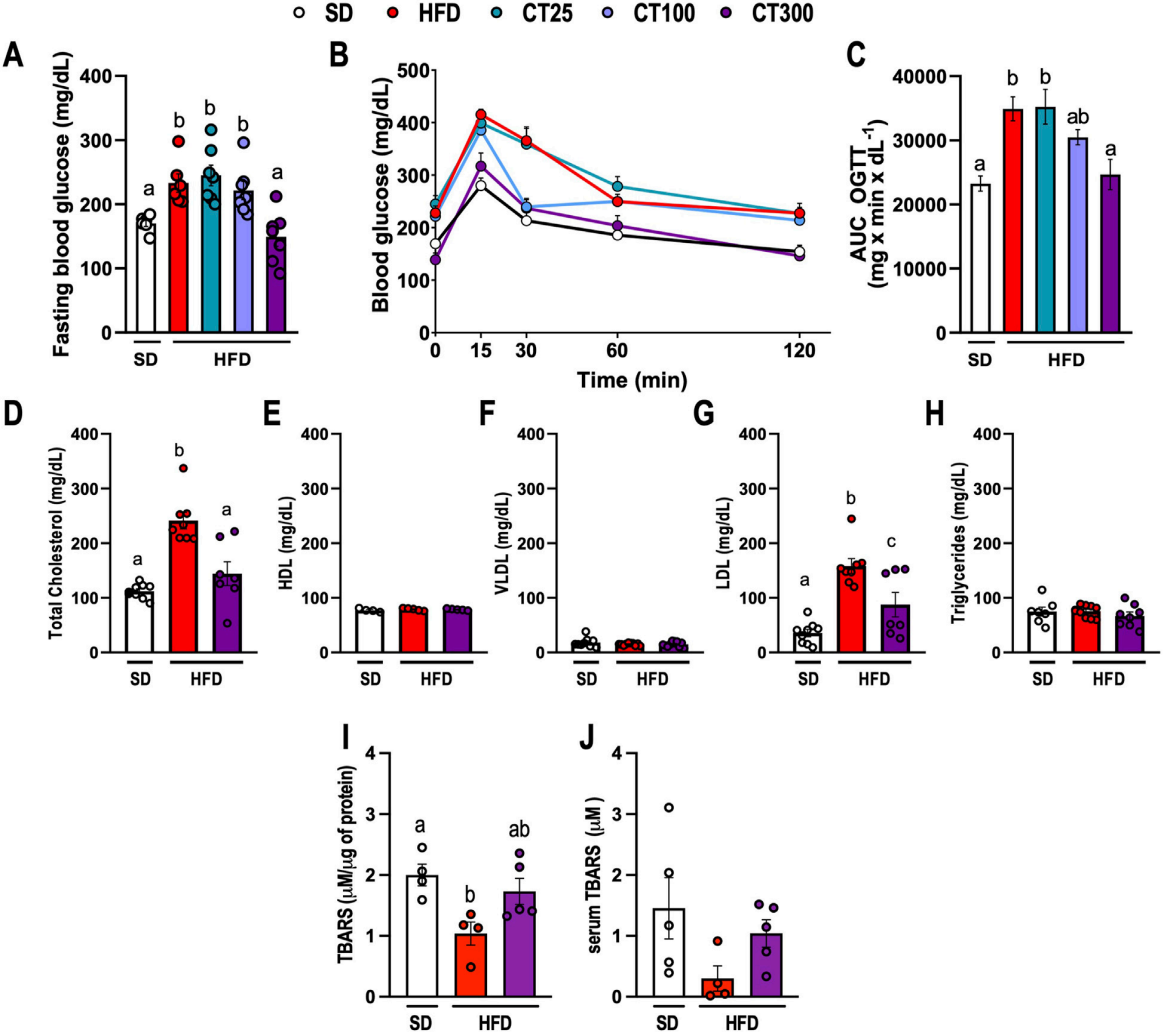
Effect of Citral (CT) treatment against high-fat diet-induced metabolic changes in glucose and lipid profile in obese C57Bl/J6 mice. **(A)** Fasting blood glucose. **(B)** Blood glucose during oral glucose tolerance test (OGTT). **(C)** Area under curve (AUC) of OGTT. Lipid profile of the serum levels of **(D)** total cholesterol, **(E)** high-density lipoprotein (HDL), **(F)** very low-density lipoprotein (VLDL), **(G)** low-density lipoprotein (LDL), and **(H)** triglycerides. TBARS levels in Epi AT **(I)** and serum **(J)**. Data are expressed as mean ± S.E.M. (n = 5–7). Different lowercase letters (e.g., “a,” “b,”, “c,” and “d”) indicate statistically significant differences among the groups (p < 0.05), with different letters representing distinct groups and shared letters indicating no significant difference. SD, standard diet; HFD, high-fat diet.

CT treatment prevented the HFD-induced metabolic alterations after obesity induction (Figure 2). HFD ingestion promoted an increase in fasting blood glucose and hyperglycemia in obese mice compared to those in the SD group (p < 0.05; Figure 2A–C). In obese mice, dyslipidemia was observed, characterized by a significant increase in serum Total Cholesterol, and LDL levels, resulting in a reduction in TBARS levels in Epi-AT tissues, but not in serum levels, which was attributable to a decrease in lipid turnover in AT tissues (p < 0.05; Figures 2D, G, I, respectively). Parameters like HDL, VLDL, and triglycerides and serum TBARS were not altered by diet (p > 0.05; Figures 2E, F, H, respectively). However, chronic treatment with CT300 prevented the elevation of blood glucose, total cholesterol, and LDL levels, concomitant with the induction of obesity by HFD. The monoterpene also prevented the reduction in TBARS levels in Epi-AT caused by HFD by exerting anti-hyperglycemic and anti-hyperlipidemic actions.

### 3.3 Citral decreased the metabolic endotoxemia and low-grade inflammation promoted by HFD-induced obesity

**FIGURE 3:**
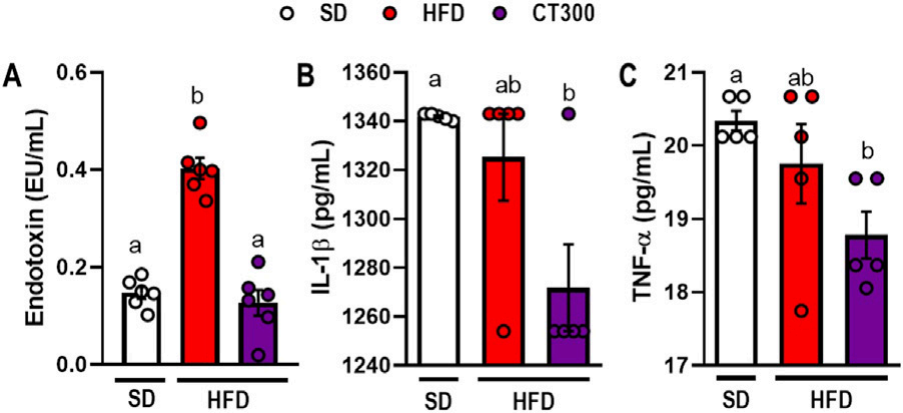
Citral (CT) treatment prevents high-fat diet-induced metabolic endotoxemia and inflammatory response in obese mice. Serum levels of **(A)** endotoxin, **(B)** IL-1β, and **(C)** TNF-α. Data are expressed as mean ± S.E.M (n = 4-5). Different lowercase letters (e.g., “a,” “b,”, “c,” and “d”) indicate statistically significant differences among the groups (p < 0.05), with different letters representing distinct groups and shared letters indicating no significant difference. SD, standard diet; HFD, high-fat diet.

HFD ingestion induced inflammatory alterations associated with metabolic endotoxemia (Figure 3). We observed an increase in endotoxin levels in obese mice compared to those in the SD group (p < 0.05; Figure 3A). Conversely, CT300 treatment exhibited an anti-inflammatory effect, preventing a systemic increase in LPS levels after 17 weeks (p < 0.05; Figure 3A). This resulted in decreased serum IL-1β, and TNF-α levels in mice, compared to the obese group (Figures 3B, C, respectively).

### 3.4 Citral protects mice against intestinal changes caused by HFD ingestion for 17 weeks

**FIGURE 4:**
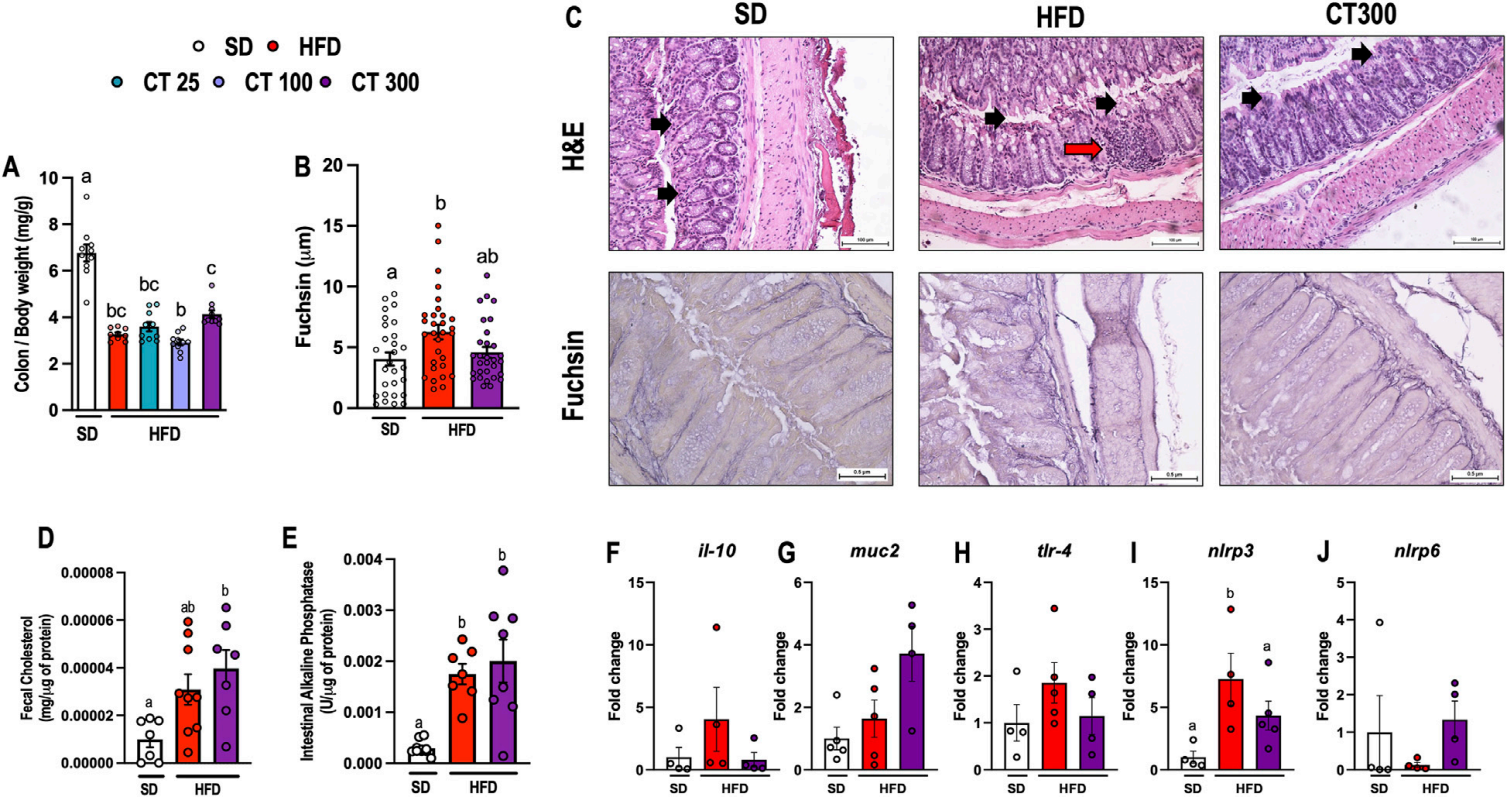
Chronic Citral (CT; 300 mg/kg) treatment impairs high-fat diet-induced colon changes in obese mice. **(A)** Relative mass of colon. **(B)** Quantification of fuschin. (n = 3 animals per group/30 microcrographs per group). **(C)** Representative histological images of H&E and fuchsin. The black arrow indicates the intestinal epithelium, and the red arrow indicates the cellular inflammatory infiltrate. **(D)** Fecal cholesterol levels. **(E)** Intestinal alkaline phosphatase activity. Gene expression of **(F)** il-10, **(G)** muc-2, **(H)** tlr-4, **(I)** nlrp3, and **(J)** nlrp6 in the colon. Data are expressed as mean ± S.E.M (n = 3–10). Different lowercase letters (e.g., “a,” “b,”, “c,” and “d”) indicate statistically significant differences among the groups (*p* < 0.05), with different letters representing distinct groups and shared letters indicating no significant difference. SD, standard diet; HFD, high-fat diet.

Intestinal alterations occur during the development of obesity (Figure 4). Mice fed an HFD showed a decrease in the relative mass of the colon compared to the SD group after 17 weeks (p < 0.05; Figure 4A). This change was associated with a significant increase in elastin deposition (p < 0.05; Figure 4B) caused by damage to the intestinal epithelium and immune cell infiltration following HFD ingestion (Figure 4C). Additionally, HFD consumption caused gut dysbiosis, which triggered an increase in intestinal alkaline phosphatase activity in obese mice compared to that in the SD group (p < 0.05; Figure 4E).

In contrast, treatment with CT300 prevented HFD-induced colonic damage in mice 17 weeks after obesity induction. Additionally, CT300 treatment promoted the preservation of relative colon mass when compared to the different dose levels (Figure 4A) and prevented the deposition of elastin and morphological changes in the colon of obese mice (Figures 4B, C). Despite the increased lipid intake from HFD, fecal total cholesterol levels did not significantly increase; however, CT300 treatment caused a significant increase in lipid excretion (p < 0.05; Figure 4D). In addition, CT300 exhibited anti-inflammatory action in the colon through the reduction of *nlrp3* gene expression when compared to the HFD group (Figure 4I). The expression of *il-10*, *muc2, tlr-4* and *nlrp6* were not changed by treatment or diet (p > 0.05 Figures 4 F-H, J, respectively).

### 3.5 Citral improves tight-junction protein levels in CMT-93 cells stimulated with LPS

**FIGURE 5:**
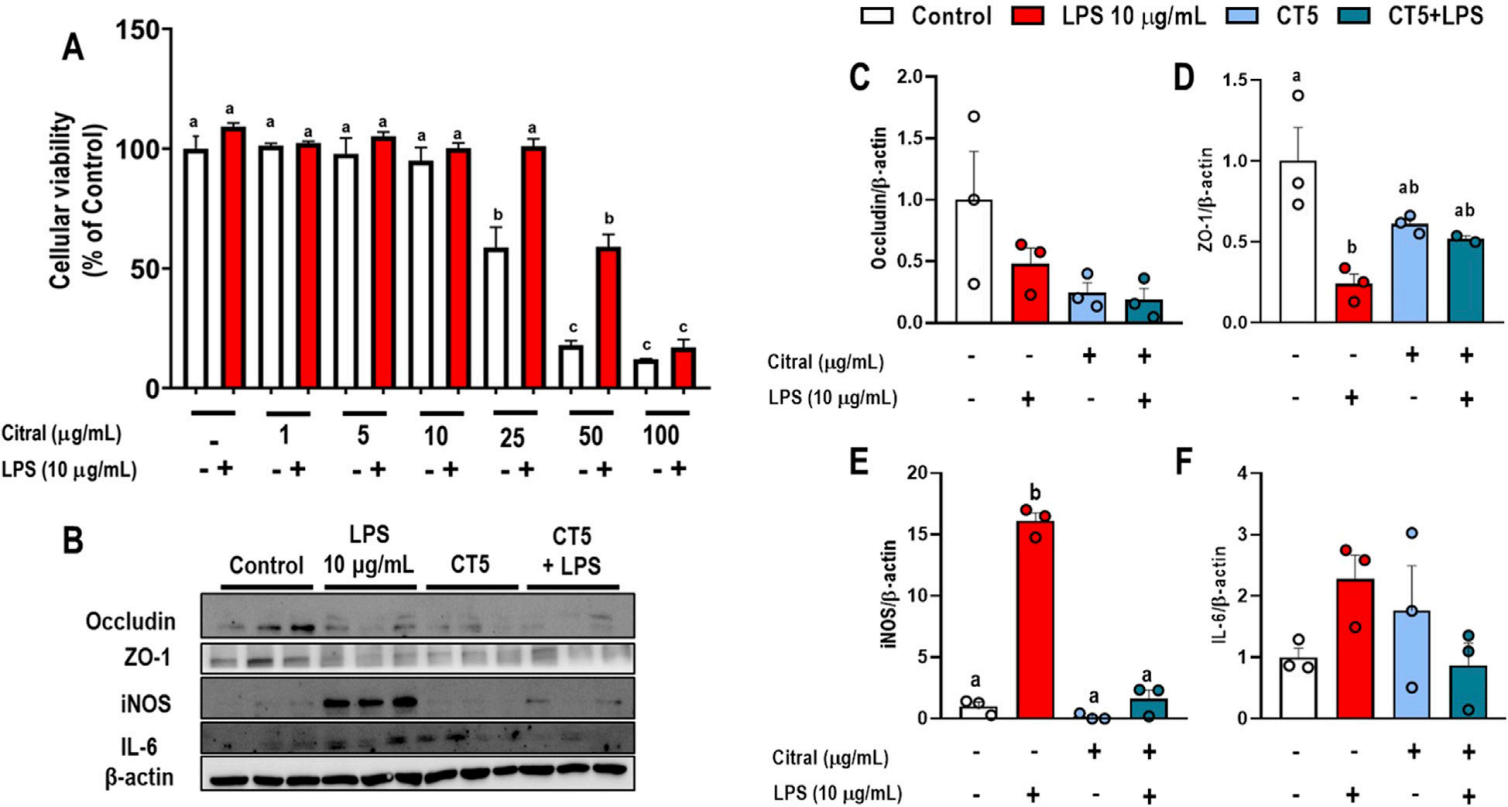
Effects of Citral (CT) in murine rectal carcinoma CMT-93 cells stimulated with LPS (10 μg/mL). **(A)** Cellular viability. **(B)** Representative Western blots images. Protein expression levels of **(C)** occludin, **(D)** ZO-1, **(E)** iNOS, and **(F)** IL-6. Data are expressed as mean ± S.E.M. (n = 3-4). Different lowercase letters (e.g., “a,” “b,”, “c,” and “d”) indicate statistically significant differences among the groups (p < 0.05), with different letters representing distinct groups and shared letters indicating no significant difference.

Cellular viability and/or possible toxic effect of different concentrations of CT after 72 h of stimulation with LPS 10 μg/mL in CMT-93 cells were investigated (Figure 5A). The cells stimulated with LPS 10 μg/mL did not alter the cellular viability, which was also observed in cells when stimulated with CT at 1, 5 and 10 μg/mL at when compared to the control group (p > 0.05). However, CT at concentrations higher than 25 μg/mL resulted in a significant progressive reduction in cellular viability compared to the control group (p < 0.05).

CMT-93 cells stimulated with CT at 5 μg/mL (CT5) prevented the impairment of the levels of proteins related to permeability and showed an anti-inflammatory effect (Figures 5B–F). Stimulation with LPS at 10 μg/mL significantly reduced ZO-1 levels (p < 0.05; Figure 5D) and increased iNOS levels (p < 0.05; Figure 5E) compared to the control group. However, CT5 and CT5+LPS did not change ZO-1 levels (p > 0.05; Figure 5D) compared to those in control and 10 μg/mL LPS treated cells. This result may be due to the reduction of the inflammatory process caused by a significant decrease in iNOS levels in CT5 and CT5+LPS treated cells compared to the control and LPS 10 μg/mL treated cells (p < 0.05). Additionally, the levels of occludin, and IL-6 were determined, however, no significant difference was observed compared to their levels in control and LPS 10 μg/mL treated cells (Figures 5C, F, respectively).

## 4 Discussion

In this study, the effect of chronic CT treatment on the metabolic and inflammatory changes associated with LPS and HFD we investigated. Our results showed that HFD-fed animals had a final 38% higher body mass than that of SD-fed animals. Thus, the HFD was responsible for the large increase in body mass and fat accumulation. However, treatment with CT300 exhibited an anti-obesogenic response that prevented an increase in body mass gain by reducing the accumulation of body fat, mainly in the AT, independent of food intake. Previous studies have shown that rats fed a hypercaloric diet manipulated for fattening with hypercaloric inputs and treated with CT (at doses of 10, 15, and 20 mg/kg) showed suppressed abdominal fat accumulation and weight gain (Modak and Mukhopadhaya, 2011). The present study also demonstrates the protective effect of the chronic CT treatment against HFD-induced metabolic changes over 17 weeks in the development of obesity in mice.

Mice fed an HFD for 17 weeks had a significant increase in serum blood glucose, total cholesterol, and LDL levels, characteristic of a metabolic disorder. Oral treatment with CT prevented the increase in serum glucose levels and lipid profiles, probably due to the reduced circulation of dietary lipids. This metabolic change is associated with an AT mass increase due to HFD ingestion after 17 weeks. The HFD caused an increase in AT mass in mice, which may be associated with a decrease in TBARS levels, a product of lipid peroxidation, in AT-Epi cells. However, these results were not observed in serum quantification, probably because of a lower rate of peroxidation in AT owing to a lower rate of lipid turnover, with greater storage and less release of lipids by the tissues. Additionally, AT plays an important role in the body by regulating lipid storage duration in adipocytes (Palacios-Marin et al., 2023; Spalding et al., 2008). Moreover, a direct relationship exists between adipocyte volume and total body mass, and the average lipid age in AT is significantly higher in overweight and obese individuals compared to individuals with lower body weight (Spalding et al., 2017). Therefore, a reduction in lipid turnover in AT may account for this difference in lipid age, due to a decrease in tissue lipolysis, consequently reducing the rate of lipid export, which contributes to a greater accumulation of fat in white AT.

Previous studies have elucidated the ability of CT to prevent the development of obesity. The monoterpene contributes to the modulation of lipid accumulation and differentiation of 3T3-L1 preadipocytes, mediated by decreased lipid uptake and increased lipolysis, resulting in a strong influence on adipocyte metabolism (Sprenger et al., 2022; Sri Devi and Ashokkumar, 2018). However, our results showed that the CT treatment prevented changes in lipid accumulation in the AT of obese animals, which may be related to their anti-obesogenic response to HFD ingestion. Additionally, CT prevented hyperlipidemia and hyperglycemia in obese mice, probably due to the intestinal changes induced by the protective action of the monoterpene against diet-induced metabolic and intestinal changes.

Metabolic endotoxemia caused by gut dysbiosis in obese individuals plays an important role in triggering low-grade chronic inflammation and metabolic dysfunction (Mohammad and Thiemermann, 2021; Thaiss et al., 2018). This systemic increase in LPS contributes to body weight gain and development of metabolic disorders in obese individuals (Cani et al., 2007; Kim et al., 2012). LPS, associated with intestinal dysbiosis, is recognized by the TLR-4 receptor which leads to the induction of insulin resistance, mainly through phosphorylation of IRS-1, the main marker of this condition in obesity (Kim et al., 2012; Saad et al., 2016). In addition, endotoxin-induced activation of inflammatory responses, including production of TNF-α and IL-1β, is associated with chronic inflammation (Liaqat et al., 2021; Deng et al., 2025). TLR-4 activation also increases iNOS expression, leading to an increase in serum NO levels, which contribute to an oxidative stress response that potentiates hyperglycemia and insulin resistance (Sugita et al., 2002). Here, we present a novel finding that has not been previously demonstrated that chronic treatment with CT reduces endotoxin levels in obese mice, suggesting that its action may play a key role in preventing the development of hyperglycemia and low-grade systemic inflammation. This preventive response may be associated with direct protection against intestinal damage caused by HFD ingestion.

The intestinal epithelium plays an indispensable function in protecting and controlling energy intake by the host, mainly through the selective passage of nutrients, water, and minerals through the intestinal mucosal barrier (Allaire et al., 2018). Protective factors involved in the control of homeostasis play an important role in the luminal space, such as the adequate expression of mucus and intestinal alkaline phosphatase, which dephosphorylates and detoxifies LPS from the gut microbiota (Ghosh et al., 2020). Thus, an imbalance in the normal function of the intestinal barrier has a significant impact not only on the gastrointestinal tract but also on the entire body. Recent studies have shown that long-term hypercaloric diets have a negative impact on gut epithelial integrity, mainly due to morphological changes and a reduction in TJ proteins caused by an inflammatory process that promotes an increase in intestinal permeability (Xie et al., 2020). An important factor in the development of obesity is altered nutrient transport through the intestinal mucosal barrier (Kawaguchi et al., 2023; Tanaka et al., 2020). Fat overnutrition promotes adaptation of the intestinal epithelium, facilitating greater absorption and digestion of compounds, resulting in greater fat accumulation and body weight (de Wit et al., 2011). In addition, HFD feeding causes a significant increase in LPS levels in the gut microbiota, which contributes to the barrier breakdown, with an abnormal architecture of the gut epithelium and an increase in cell death in the villi and crypts (Leyderman et al., 2023). This scenario leads to increased permeability and inflammatory processes in the intestinal epithelium (Mohammad and Thiemermann, 2021). Therefore, the maintenance of gut integrity is a critical factor in the development of metabolic endotoxemia. A correlation has also been observed between increased intestinal alkaline phosphatase activity and dysbiosis (Estaki et al., 2014). This condition is characterized by the elimination of endotoxins and other bacterial compounds that maintain intestinal homeostasis. In this study, the protective effect of CT against damage caused by chronic exposure to HFD in the gut epithelium of mice for over 17 weeks was demonstrated. The monoterpene prevents the increase in *nrlp3* expression, a proinflammatory gene, and morphological changes induced by HFD. Additionally, CT-treated obese mice exhibit enhanced lipid fecal elimination, suggesting a potential mechanism of action against the development of obesity.

Thus, CT showed an important protective effect against HFD-induced obesity by preventing intestinal inflammation associated with diet and gut dysbiosis. This effect may be mediated by a reduction in the expression of *nlrp3* and iNOS, as elucidated in obese C57Bl/J6 mice and CMT-93 cells stimulated with LPS. Although previous studies have shown the same anti-inflammatory effect of CT, the potential preventive action of the monoterpene against HFD-induced metabolic endotoxemia from intestinal changes was not demonstrated. In the porcine jejunum epithelial cell line (IPEC-J2), CT decreased the inflammatory response mediated by peptidoglycan, improved ZO-1 and claudin levels, and increased epithelial resistance, ensuring intestinal integrity and functionality (Li et al., 2022). The monoterpene also exerts antioxidant effects against aspirin-induced damage in rat small intestine epithelial cells. This response is mediated by the modulation of glutathione and superoxide dismutase, which results in a significant decrease in MDA levels, an important marker of cellular membrane lipid peroxidation (Bouzenna et al., 2017). However, CT at 25 μg/mL showed a negative effect related to the cytotoxic effect, caused by DNA damage resulting in decreased cellular migration and viability (Souza et al., 2020). Although this effect is controversial, as different lines and models demonstrate the beneficial effects of the compound, our results show that CT promoted pharmacological activity against HFD and LPS stimulation. Thus, we must consider that different conditions and experimental models may yield different responses, which should be investigated further.

Despite the important findings on the role of CT as a compound with an anti-obesity activity in mice, further studies are still needed to determine its effects on other systems that regulate body weight, such as muscle tissue and the central nervous system (Della Guardia and Shin, 2024). The present study did not investigate the effect of CT on increased permeability and changes in the microbiota due to the reduced number of samples per group, which limited the analyses performed. However, future studies are required to elucidate the role of CT in gut dysbiosis and the production of short-chain fatty acids (SCFAs) in obese individuals. It has been established that the HFD-induced changes, both intestinal and systemic, result from alterations in the gut microbiota with changes in the fecal microbiome leading to an increase in intestinal permeability (Sagkan-Ozturk and Arpaci, 2022). Additionally, a differential production of SCFAs in obesity also influences inflammatory and metabolic conditions (Leyderman et al., 2023). These factors become potential therapeutic targets that may be involved in the anti-obesity action of the monoterpene.

In summary, the results presented in this study demonstrate the anti-hyperglycemic, anti-lipidemic, and anti-inflammatory effects of CT against HFD-related obesity in mice. Chronic treatment with CT alleviated metabolic dysfunction mediated by its protective action in the gut epithelium and increased lipid elimination in feces. The anti-obesogenic effect of this monoterpene is mediated by intestinal modulation, demonstrating that it is a potential drug that can prevent systemic abnormalities and act against the development of diet-related obesity.

## Data Availability

The raw data supporting the conclusions of this article will be made available by the authors, without undue reservation.
